# Past and Present Role of Neurosurgical Interventions in the Management of Psychiatric Disorders: A Literature Review on the Evolution of Psychosurgery

**DOI:** 10.7759/cureus.79022

**Published:** 2025-02-14

**Authors:** Naveen Arunachalam Sakthiyendran, Venkata Jaswanth Padala, Melinda Seide, Jia Whei See, Nagma Sabu, Asmita Sharma, Mohammed T Silat, Kabeer Katariya, Sonali Chauhan, Urooj Fatima

**Affiliations:** 1 Neurological Surgery, Boston University School of Medicine, Boston, USA; 2 Internal Medicine, GSL Medical College, Rajamahendravaram, IND; 3 Internal Medicine, St. George's University School of Medicine, St. George's, GRD; 4 General Medicine, Universitas Sriwijaya, Palembang, IDN; 5 Surgery, Jonelta Foundation School of Medicine University of Perpetual Help System Dalta, Metro Manila, Las Piñas, PHL; 6 Oncology/Otorhinolaryngology, Jorhat Medical College and Hospital, Hamirpur, IND; 7 Medicine, Dow International Medical College, Karachi, PAK; 8 Psychiatry, Shyam Shah Medical College, Rewa, IND; 9 Neurology, John F. Kennedy University School of Medicine, Willemstad, CUW; 10 General Practice, Dow University of Health Sciences, Civil Hospital Karachi, Karachi, PAK

**Keywords:** ablative surgeries, deep brain stimulation (dbs), mental health interventions, neuromodulation therapies, obsessive-compulsive disorder (ocd), psychiatric neurosurgery, transcranial magnetic stimulation (tms), treatment-resistant disorders

## Abstract

Despite advancements in psychiatric treatments, many patients with treatment-resistant disorders are turning to neurosurgical interventions. These include neuromodulation-based surgeries such as deep brain stimulation (DBS) and ablative surgeries such as cingulotomy, offering relief for severe conditions such as post-traumatic stress disorder (PTSD), depression, schizophrenia, obsessive-compulsive disorder (OCD), anxiety, and substance use disorder. While "psychosurgery" has sparked debate due to concerns about patient well-being, recent studies indicate promising symptom improvement rates across various psychiatric conditions while also demonstrating overall safety. Neuromodulation techniques, such as DBS, transcranial magnetic stimulation (TMS), and electroconvulsive therapy (ECT), have evolved in regard to their sensitivity and their ability to target specific brain regions to alleviate psychiatric symptoms. Despite their benefits, these therapies have been shown to elicit side effects such as memory loss and seizures in patients, which has sparked controversy in the use of this technology across clinicians and patients. Ablative therapies, on the other hand, are concerning for being overly invasive in their approach toward psychiatric care. Despite the stigma associated with these neurosurgical interventions for psychiatric care, these procedures often remain a last resort for many patients, highlighting the need for continued research to improve these treatments and expand options for those in need. In this narrative review, we examine the current literature to elicit an understanding of neurosurgical history in regard to psychiatric disorder treatment and its implications for clinical practice.

## Introduction and background

Psychosurgery, the concept of physically altering the brain to alleviate disorders of the mind, has an ancient and fascinating history spanning from primitive practices in the Stone Age to the cutting-edge technology of the 21st century. The earliest recorded psychosurgical procedure is trephination, which was practiced by prehistoric humans due to the perception that it could release evil spirits from one's head by a scalp incision and drilling a hole in the skull. Trephination continued as a practice from 5000 BC up until the Renaissance period. The decline of trephination as a psychiatric intervention during the 18th and 19th centuries, due to the adjacent development of antipsychotic medications, also saw the rise of the idea that distinct areas of the brain are responsible for specific functions of the body. Studies during this period by Wernicke and Broca laid the foundations of modern psychosurgery [1,2].

There have been various advancements and improvements in pharmacological treatments for psychiatric disorders in recent years, such as obsessive-compulsive disorder (OCD), depression, anxiety, and schizophrenia. However, it has been shown that many patients remain resistant to treatment [3]. To address these limitations, neurosurgical interventions have grown in popularity and are considered especially in patients who face psychiatric disorders resistant to pharmaceutical therapy [3,4].

Modern psychosurgery encompasses two primary approaches: ablative neurosurgery and neuromodulation-based procedures. Ablative techniques, such as anterior cingulotomy and subcaudate tractotomy (SCT), involve targeted lesioning of neural circuits implicated in psychiatric disorders [1]. In contrast, neuromodulation techniques, including deep brain stimulation (DBS), transcranial magnetic stimulation (TMS), and electroconvulsive therapy (ECT), modulate dysfunctional neural pathways without causing permanent damage [3,4]. These techniques have gained traction as safer and more effective alternatives, offering a reversible and adjustable means of intervention.

Although novel neurosurgical interventions are becoming more prevalent in psychiatric treatment paradigms, this was not the case throughout most of history. Given the major side effects and complications faced by patients, such as worsening depression, suicidal ideation, infection, and seizures, and the lack of research, consideration, and oversight, neurosurgery was frowned upon [3]. In the present era, neurosurgical interventions have been improving and transforming toward a more educated direction and are practiced with a more complete consideration of patient safety owing to the development of more advanced operative and diagnostic tools [4].

In this narrative review of current literature, we discuss the relationship between various neurosurgical interventions and the efficacy of these modalities in symptom management of various psychiatric disorders. The objective of this study is to share the latest findings, clinical objectives, and achievements to contribute to a breakthrough of knowledge in this topic. Additionally, this review also aims to find gaps in current knowledge to offer insights on improving the approach toward psychiatric patient care and the potential use of neurosurgical interventions.

## Review

Epidemiology

In the United States and many other parts of the world, psychiatric illnesses are a major challenge to combat. Statistically speaking, approximately 25% of patients in the United States are diagnosed with psychiatric illnesses, and about 20%-60% of patients with psychiatric disorders are battling with pharmaceutical treatment resistance [5]. Additionally, in 2019, $106.5 billion was spent to treat psychiatric disorders such as anxiety, depression, bipolar disorder, and other mental and neurodevelopmental illnesses in adults 18 years and older [6]. These disorders tend to dramatically affect patients' daily living, interpersonal relationships, and finances and lower the overall quality of life [5,7-9]. Medication and behavioral therapy fail to alleviate symptoms in around 10%-25% of people with mental illness [10,11]. It is very improbable that 20%-30% of people with schizophrenia would have healthy lives due to their resistance to antipsychotic medication [10,11]. It is possible to derive similar conclusions when dealing with resistant individuals who suffer from other mental health issues. One percent of young women suffer from anorexia nervosa (AN), a mental illness that has the highest mortality rate of any mental illness [6].

About 10,000 leucotomies were carried out in the United States and the United Kingdom before psychiatric medications were available [12]. This number had increased significantly, and there were over 60,000 operations performed a few years later [13,14]. Within the first year of chlorpromazine's launch in 1954, the medicine was administered to almost two million patients [13]. The yearly number of ablative procedures performed in the United States has dropped below 15 in recent years [12].

Etiology

The understanding of mental disorders remains a challenge, even if diagnostic tool developments have improved physicians' knowledge of the brain and its activities in recent decades. The origins of many mental health disorders are complex, according to medical experts, and both genetics and environmental factors play a significant role [5,7]. While the exact cause of schizophrenia is still unknown, genetic factors may account for around 80% of cases [12]. Some of the environmental variables that have been linked to the development of schizophrenia in early life include maternal infection, malnutrition, and urbanization. Similarly, structured narratives have been used to shed light on OCD, anxiety disorders, and depression [7]. The susceptibility of anorexia nervosa (AN) to hereditary factors is significant [15].

Pathology

New diagnostic tools and the ability to combine data from several modalities have improved our understanding of the brain's anatomy and physiology, allowing for more precise localization of neurological diseases. Electroencephalography (EEG), functional magnetic resonance imaging (fMRI), transcranial magnetic stimulation, and positron emission tomography are several diagnostic methods [16-18]. These diagnostic methods provide a stronger foundation for targeted neuromodulation surgery and a better understanding of brain processes in certain mental illnesses.

According to research, abnormal connections between the brain's cortex, striatum, and thalamus are the cause of obsessive-compulsive disorder. The corticostriatal pathway, where the motor cortex and other cortical regions send projections to the striatum, is crucial for motor control, habit formation, and cognitive functions [19]. In OCD, dysregulation within this pathway, along with altered interactions between the striatum and thalamus, can lead to maladaptive behaviors. The striatum sends projections to the thalamus through the indirect pathway, involving structures such as the globus pallidus externus and subthalamic nucleus, which modulate thalamic output to the cortex [19]. These thalamocortical loops, which are important for integrating sensory and motor information, may be disrupted in OCD, contributing to the characteristic compulsive behaviors [7]. Additionally, structural abnormalities, including reduced integrity in the genu of the corpus callosum, have been observed in both schizophrenia and depression, which disrupt interhemispheric communication between the frontal lobes [20]. These changes are linked to impaired cognitive and emotional regulation, with altered functional connectivity contributing to symptoms such as cognitive deficits in schizophrenia and emotional dysregulation in depression. On the other hand, research suggests that anxiety disorders may have their roots in the medial cingulate cortex, a key region involved in emotional regulation and cognitive control [21]. Altered activity and connectivity in the mid-cingulate cortex (MCC), particularly in its role in threat detection and response inhibition, are thought to contribute to the heightened emotional sensitivity and impaired coping mechanisms seen in anxiety disorders [22,23]. An established target for neuromodulation in obsessive-compulsive personality disorder (OCPD) and depression is the inferior thalamic peduncle (ITP), which connects the thalamus to the orbitofrontal cortex. Research suggests that modulation of the ITP can help restore balance in neural circuits related to compulsive behaviors in OCPD and depressive symptoms, with studies highlighting its potential for improving treatment outcomes [24].

In treating severe psychiatric disorders unresponsive to standard therapies such as psychotherapy, pharmacotherapy, and electroconvulsive therapy, surgical intervention becomes a consideration. Common procedures such as cingulotomy, subcaudate tractotomy (SCT), capsulotomy, and limbic leucotomy (LL) show response rates ranging from 35% to 65%, with modern techniques reducing complications. However, debate persists over the optimal surgical approach. Conditions such as treatment-refractory major affective disorders, obsessive-compulsive disorder, and chronic anxiety states are often targeted. Surgical intervention should be part of a comprehensive treatment plan and conducted by a specialized multidisciplinary team including neurologists, neurosurgeons, and psychiatrists. Despite being underutilized, surgical intervention remains a viable option for select patients with debilitating psychiatric conditions [25].

**Figure 1:**
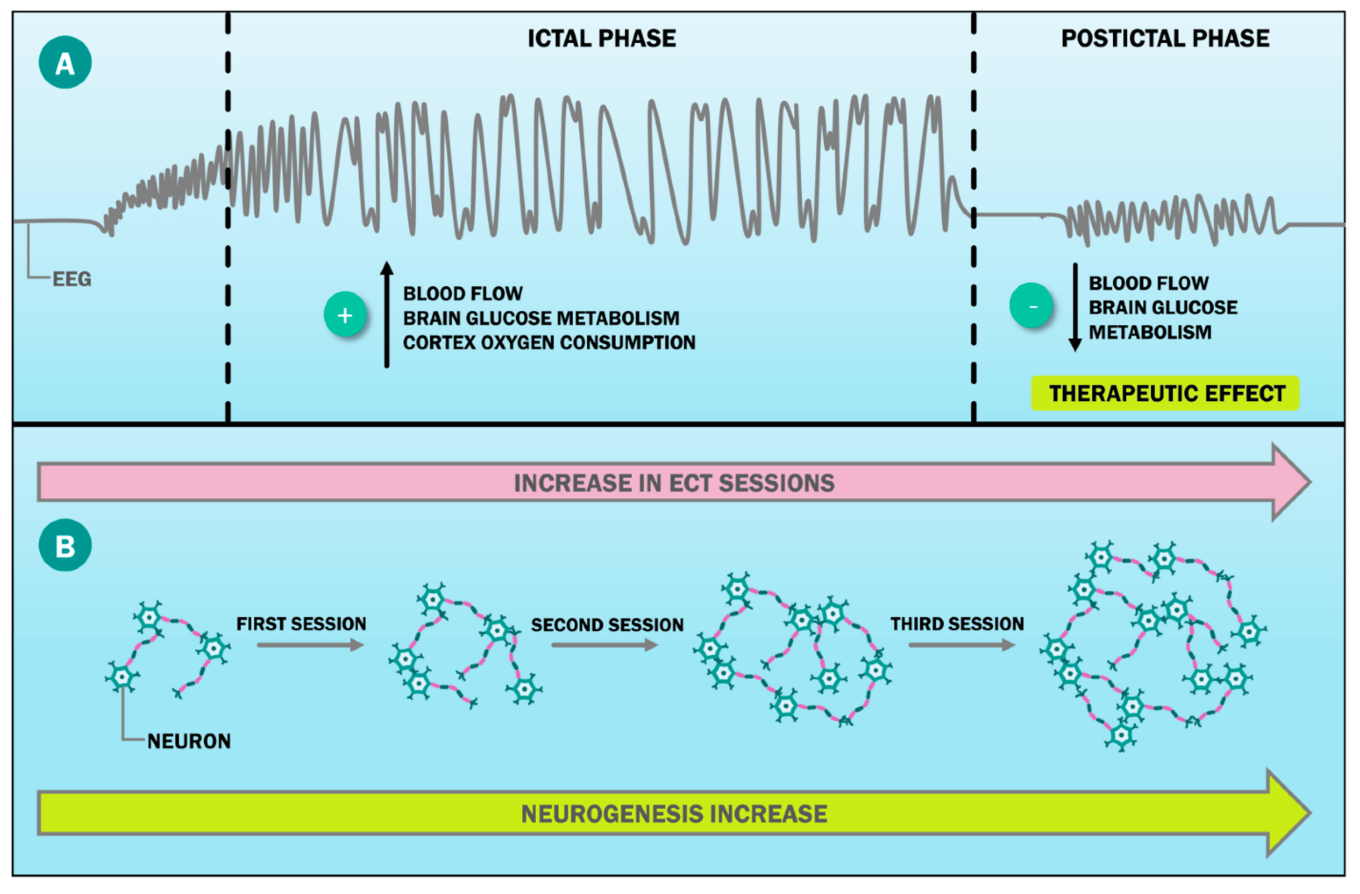
Neurobiological effects of ECT ECT produces therapeutic neurobiological effects, including (A) alterations in brain cortex metabolism, which vary across different ECT phases (these changes are detectable through EEG, with the postictal phase being most strongly linked to ECT's therapeutic impact), and (B) stimulation of hippocampal neurogenesis, which occurs in a dose-dependent manner. ECT: electroconvulsive therapy, EEG: electroencephalography Reproduced with permission from Davidson et al.: Examining cognitive change in magnetic resonance-guided focused ultrasound capsulotomy for psychiatric illness. Transl Psychiatry. 2020, 10:397. 10.1038/s41398-020-01072-1 [39]

In treating psychiatric disorders, clinicians typically resort to a combination of therapies, including medication adjustment, psychotherapy, and somatic treatments such as electroconvulsive therapy (ECT) [25]. However, when patients fail to respond adequately, augmentation strategies are often considered. These typically involve adding another medication or therapy to the existing regimen. While conventionally introduced later in treatment, there is growing interest in early augmentation, aiming for faster symptom relief and functional recovery. For instance, early use of repetitive transcranial magnetic stimulation (rTMS) has shown promise in reducing depressive symptoms [26]. Similarly, electroconvulsive therapy has been favored for augmenting pharmacotherapy in conditions such as schizophrenia [27]. Despite concerns about its side effects, recent evidence suggests minimal cognitive impact and potential cognitive improvement with ECT [28,29]. Additionally, studies suggest early intervention, including ECT, may improve outcomes in first-episode psychosis and shorten untreated illness duration in depression [30,31]. Neuromodulation techniques such as transcranial direct current stimulation (tDCS) have also shown efficacy as augmentation strategies, particularly in reducing symptom severity [32,33]. While early augmentation presents challenges such as increased costs and potential side effects, it holds promise for hastening symptom relief and improving overall outcomes. Extensive research is needed to better understand the role of early augmentation in psychiatric care [34]. Some have postulated that ECT's neurobiological effects stem from its modulation of cortical activity, particularly in the prefrontal and temporal lobes [34]. Recent advances in imaging have made it possible to identify three distinct phases of ECT. During the first, known as the ictal phase, the cortex increases blood flow, glucose metabolism, and oxygen intake. The post-ictal phase comes in at number two, while the inter-ictal period comes in at number three. Most importantly, during the post-ictal phase, brain glucose metabolism and blood flow decrease, both of which are connected with ECT's effectiveness in treating depression (Figure 1A) [35]. Similarly, Oltedal et al. [34] and Cao et al. [35] are among the writers who have postulated that ECT increases neurogenesis in multiple subfields, including the dentate gyrus at the granular and molecular cell layers, the subiculum, and the cornus ammonis. The stronger the ECT dosage, the larger the volume rise, as seen in Figure 1B [36-38].

Patient outcomes following neurosurgical interventions

Treatment-Resistant Depression (TRD)

Neurosurgical interventions have led to a variety of results in patients facing various psychiatric pathologies [39-48]. In patients with treatment-resistant depression (TRD), studies have shown that daily transcranial magnetic stimulation (TMS) over the left dorsolateral prefrontal cortex (DLPFC) has over a 50% improvement in their baseline when using the Hamilton Rating Scale for Depression scores [49]. Due to the success of TMS in treating TRD, the procedure is used worldwide and is generally tolerated well by patients. On the contrary, TMS requires multiple sessions, which oftentimes makes it difficult to implement. For instance, when initially starting TMS, patients are required to have active treatment sessions that last 40-60 minutes for five days in a week for approximately 3-6 weeks. There have been attempts to adjust the TMS protocol in the last couple of decades, but they have not been successful [50]. Recently, a new protocol from Stanford University known as the SAINT protocol has been found to achieve faster remission following TMS therapy, an indication that research is going in the right direction [51].

Furthermore, epidural cortical stimulation (EpCS) has been explored as a potential TRD treatment option. One study used EpCS as an adjunctive therapy for patients who failed previous treatment modalities such as vagus nerve stimulation (VNS), electroconvulsive therapy, and TMS. During EpCS, the patients had bilateral paddle leads placed epidurally over Brodmann area 10, which is the frontopolar prefrontal cortex, and area 46, which is the dorsolateral prefrontal cortex. This method is potentially safer than DBS because DBS requires the insertion of leads directly into the brain tissue [52]. The study consisted of six participants originally, but one patient decided not to continue prior to implantation, so in total, there were only five participants. All participants were unemployed, and three of them were on disability. It had four women and one man; three women had MDD, and two had bipolar affective disorder I, depressed type. Prior to the insertion of the leads, the patients scored 20 or more on the Hamilton Rating Scale for Depression and post the 2-3-week implantation time frame. After the leads were inserted, the patients recovered for 2-3 weeks, and they received a 20-minute psychological screening prior to the leads being activated [52]. The patients had to follow up for seven months, and it was found that the patients had over 50% improvement on average in their depression compared to their status preimplantation, and 80% of the cohort obtained remission. Remission for these patients was maintained at three and five years post-EpCS, which makes EpCS a great potential treatment modality [53].

Deep brain stimulation (DBS) is another treatment modality for TRD. It is invasive and involves the insertion of intracranial leads stereotactically [13]. In DBS that targets the nucleus accumbens, approximately 40%-45% of the patients showed over a 50% decrease in their depression symptoms. For patients who received ventral striatum/capsule DBS, studies at 12 months found a 53% response rate, and at around 37 months, a 71 % response rate. Furthermore, subcallosal cingulate (SCC) cortex DBS studies showed a response rate of over 60% [53].

There was a multicenter follow-up trial that demonstrated a 29% response rate at 12 months, while a follow-up single-blinded study showed a response rate of 98% and a rate of remission of 58% [53]. The difference in these follow-up trials could be due to a higher stimulation intensity of 6-10 mA, while the multicenter trial utilized an intensity of 5.2 mA. These follow-up trial results then led to the BROADEN trial, which was a randomized SCC DBS multicenter trial [53]. The patients were divided into two groups, had bilateral SCC implantation, and received DBS treatment for six months or sham treatment. At six months, no differences were noted, and the study was dismantled, but they did follow the participants for 2-8 years and found that the response rate rose to 81% and the remission rate rose to 54%. These results thus demonstrate the potential of DBS and the need to possibly take an individualized approach due to the heterogeneity of psychiatric pathologies. Furthermore, although DBS has potential therapeutic use for TRD, its results have not been reproduced in randomized clinical trials. In open-label investigations, DBS has shown some potentially promising results [54].

Lastly, some studies have tested magnetic resonance-guided focused ultrasound (MRgFUS) anterior capsulotomy (AC), a procedure that involves lesioning the white matter of the anterior internal capsule, on treatment-resistant depression [55]. There has been a reluctance to use invasive procedures such as anterior capsulotomy because of the potential damage to cognition. The MRgFUS procedure uses ultrasonic waves to create lesions, which allows clinicians to perform anterior capsulotomy with a lower risk of infection and hemorrhage, with less ionizing radiation, thus making the procedure a lot safer. They found that at six and 12 months, the memory, executive function, and processing of the patients improved. Depressive symptoms in these patients were assessed using the Hamilton Rating Scale for Depression. At six months, none of the patients showed a 50% or more increase from baseline. At 12 months, only one patient showed a 50% or more increase from the baseline. They also found that the patient's clinical symptoms improved when there was improvement in self-reported measures of disinhibition and apathy. Overall, it was concluded that this treatment method is safe for these patients and does not have a dire impact on their cognition [55].

Alcohol Use Disorder (AUD)

Neurosurgical techniques have also been used to address alcohol use disorder. Both invasive and non-invasive repetitive transcranial stimulation that focus on the prefrontal cortical areas as well as the basal ganglia systems are tolerated by patients and are deemed safe. They also show positive potential in the management of alcohol craving and consumption in patients [56]. The best modality for severe life-threatening forms of AUD is invasive deep brain stimulation, while non-invasive DBS (rTMS and transcranial direct current stimulation (tDCS)) should be used for less severe forms. To ensure that deep brain stimulation is efficient in the AUD patient population, it is important that the patients are characterized well in regard to their clinical characteristics such as their cravings and consumption levels, which will allow more reliable comparisons between studies. Also, outcome measures and the criteria used in evaluation need to be made clear, and cravings for alcohol should be the criteria used to determine the efficacy of the trial. Additionally, finding a method that is reliable to determine baseline cravings and post-treatment cravings is essential to make comparisons across studies. Lastly, it has been difficult to determine the main target in neuromodulation for AUD, so it is important to find a way to choose adequate targets such as the dorsolateral prefrontal cortex (DLPFC), the dorsal striatum, or another brain region [56].

Post-traumatic Stress Disorder (PTSD)

In patients suffering from PTSD, different studies have been conducted to see the therapeutic effects of different neuromodulation techniques on the pathology. One of the techniques is transcranial magnetic stimulation, which is non-invasive, and studies have shown that it reduces PTSD symptoms, especially when a lower frequency stimulation of one or less is used. The studies were conducted on patients with various types of trauma, such as sexual violence, vehicle accidents, and combat. The patients were also mostly male, and their ages ranged from 18 to 75 years old [57].

Another method used to treat PTSD was the dorsolateral prefrontal cortex stimulation. What researchers found was that patients had a larger therapeutic effect when treated with right DLPFC stimulation instead of left DLPFC, and no changes were observed when researchers compared right DLPFC to bilateral DLPFC [57].

**Figure 2:**
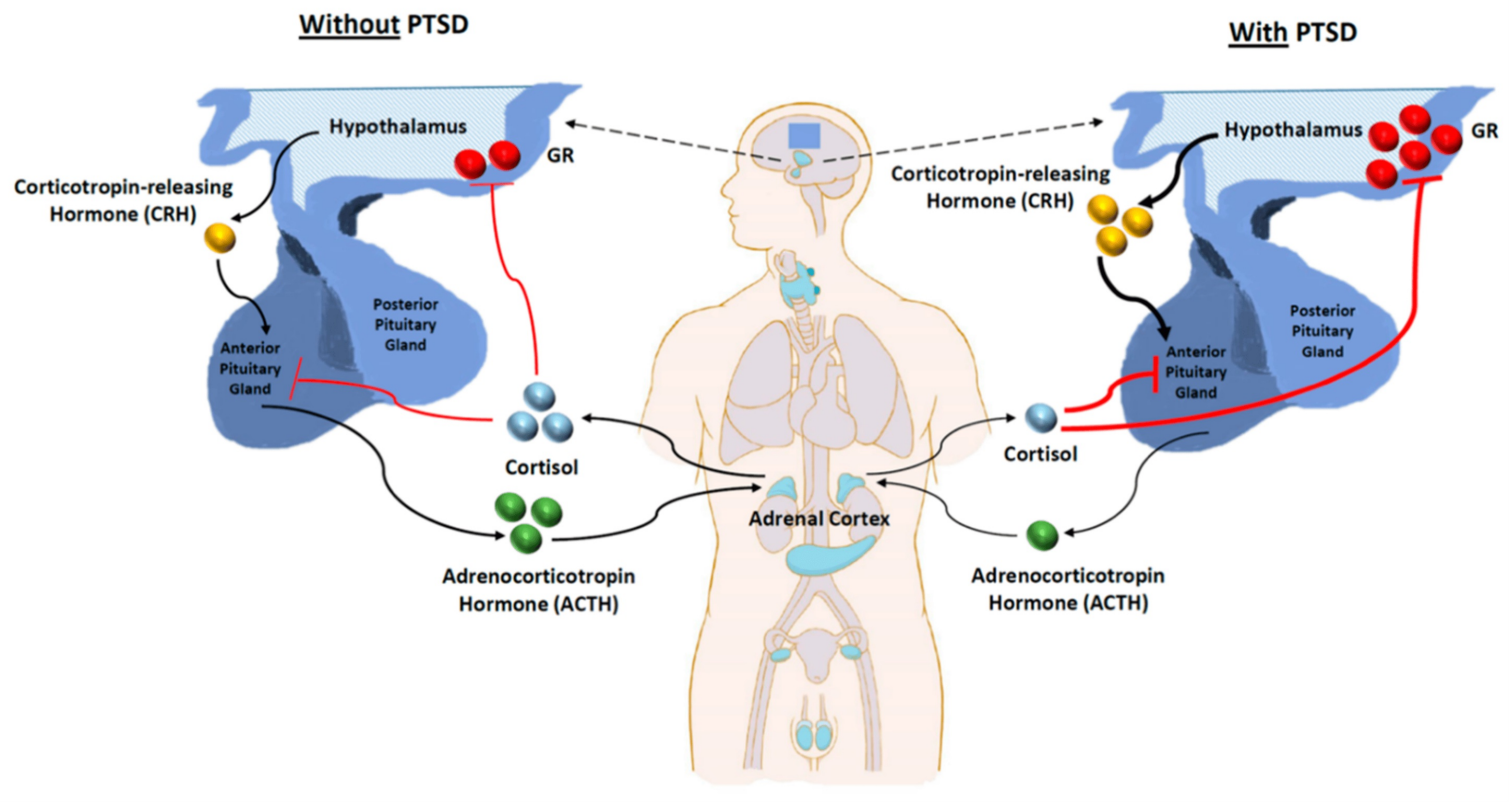
Basal HPA axis activity in both PTSD and non-PTSD cases The thicker black line represents an increase in CRH released from the hypothalamus in PTSD. With PTSD, there is a reduction in ACTH released from the anterior pituitary and, by extension, cortisol released from the adrenal cortex. Thick red lines indicate that the inhibitory effect of cortisol on the HPA axis is amplified in PTSD. PTSD: post-traumatic stress disorder, HPA: hypothalamic-pituitary-adrenal, CRH: corticotropin-releasing hormone, ACTH: adrenocorticotropic hormone Reproduced with permission from Tavakoli et al.: Literature review of the efficacy of repetitive transcranial magnetic stimulation on epilepsy. Iran J Child Neurol. 2023, 17:9-28. 10.22037/ijcn.v17i2.38752 [58]

Furthermore, it was found that when TMS was combined with psychotherapy, the patients experienced significant improvement [43]. The TMS patients also had very mild side effects such as insomnia, headache, and mood disturbances, which were addressed with pharmacotherapy [44,52]. Only in four instances, the patients experienced severe effects such as homicidal ideation, subconjunctival hemorrhage (the patient had a history of ocular disease), and generalized tonic-clonic seizures. Additionally, transcranial direct current stimulation has also shown signs of clinical efficacy, but there are fewer studies that have focused on this technique's impact on PTSD (Figure 2) [43,44].

A pilot study from 2015 found that tDCS has a positive impact on emotional and cognitive performance [53]. Another study assessed ventromedial prefrontal cortex tDCS and found it to be more effective in extinction consolidation than when combined with extinction learning [53]. Furthermore, with tDCS combined with DLPFC, it was found that patients experienced a reduction in their anxiety, depression, and PTSD symptoms after their treatment was over and when followed up one month later [4]. The tDCS studies recruited only patients who obtained PTSD from combat, and most of them were men between the ages of 20 and 69 who also had other comorbidities such as anxiety disorder and depression [53].

Lastly, deep brain stimulation, which has been used mainly for movement disorders, was used to treat combat-acquired PTSD in a 48-year-old male patient. He received bilateral DBS to his basolateral amygdala, which resulted in a 35% symptomatic improvement after receiving this treatment for eight months [58,59].

Eating Disorders

Eating disorders such as anorexia nervosa have also been a target of neuromodulation studies. It was found that DBS and other types of ablation demonstrate possible efficacy, but the current studies used small sample sizes and a short follow-up period [60]. Non-invasive methods such as tDCS and rTMS have shown a variety of results, which is due to the inability to generalize data since the studies had a range of methodologies such as case series and open-label studies [60]. Also, these studies focused on different areas in the brain and used different intensities during the treatment regimen [60]. Additionally, the patient characteristics varied across studies, such as their ages, previous treatment history, and illness duration.

In regard to weight changes, studies showed a large increase in weight with stereotactic radiofrequency (RF) ablation, but the issue is that these studies did not follow the standardized guidelines when they selected patients for neurosurgical procedures [61]. Furthermore, these studies utilized adolescents and young adults who did not have severe anorexia nervosa and who did not exhaust other psychosocial treatment modalities prior to them doing the neurosurgical option [61]. Lastly, the studies did not document psychological symptom outcomes in these neurosurgical trials, despite the fact that patients with anorexia nervosa who pursue rapid weight loss also tend to have inhibition of their appetite and phobia related to anxiety [61]. More research is needed to obtain more reliable statistics for the use of these techniques in patients with anorexia.

Obsessive-Compulsive Disorder

Another pathology that has been targeted by neurosurgical modulation is obsessive-compulsive disorder (OCD). In particular, deep brain stimulation has been used in clinical studies to address OCD. DBS is usually conducted in a continuous fashion, but it can be programmed to provide stimulation at certain intervals, which is the cyclic method [62].

A study explored the experiences of individuals with obsessive-compulsive disorder (OCD) following a positive response to deep brain stimulation (DBS). By integrating preexisting cognitive models of self-constructs and dysfunctional beliefs, new insights into the rich, phenomenological nature of DBS-assisted OCD recovery were offered. Additionally, caregivers provided their perspectives, and the challenges faced during the recovery process were discussed. The findings highlighted the importance of considering patient education, DBS effectiveness evaluation, and complementary psychotherapy models. A comprehensive, patient-specific, multidisciplinary adjunct program is essential for accurately assessing the effectiveness of DBS in treatment [62].

Furthermore, there was a study that observed the impact of capsulotomy, limbic leucotomy, and cingulotomy in treating OCD [63]. A review on observational studies that focused on the effect of capsulotomy on OCD at 12-month follow-up found that the average reduction in symptoms using the Yale-Brown Obsessive Compulsive Scale (Y-BOCS) score and the full response rate (35% or more reduction on the Y-BOCS) were 37% and 41% [62]. A meta-analysis that focused on the impact of neuroablation on OCD showed that at the last follow-up, the response rate was 55%. The observed response rates (35% or more reduction on the Y-BOCS) for cingulotomy, limbic leucotomy, and capsulotomy were 36%, 47%, and 59%, although there was some heterogeneity between the surgical groups, and the differences were not significant. The most common adverse effects reported by the patients were cognitive deficits, behavioral problems, and postoperative headaches, although these were mild and transient [63].

Safety and monitoring of neurosurgical interventions of psychiatric disorders

When considering surgical interventions for psychiatric disorders, the discussion of patient safety and ethical consensus becomes increasingly crucial. One important ethical consideration is obtaining informed consent, particularly in patients with psychosis or severe mental health issues. These individuals may have impaired capacity to fully comprehend the nature of the procedure, its risks, and potential outcomes, which raises concerns about their ability to make autonomous decisions. Special attention must be given to ensuring that patients are adequately informed, not only through clear communication but also by assessing their understanding and ability to consent. Additionally, the involvement of family members, caregivers, or mental health professionals in the decision-making process may be necessary to ensure that the patient's well-being and rights are fully protected.

While medical management of psychiatric disorders through the use of pharmaceuticals and psychotherapy has been relatively well studied, the role of neurosurgical management has been considered experimental and of more recent development (especially surgeries such as deep brain stimulation) [64]. In addition, with the historical lack of precautions and high complication risk associated with psychosurgery, there is a need for robust research to solidify neurosurgery's reputation in the treatment of psychiatric disorders. However, due to the limitations of certain drug compounds and psychiatric treatments in tackling conditions such as drug-resistant epilepsy and the promising potential of surgical treatments in achieving neuromodulation, there is a rising consideration of surgical alternatives [64].

When discussing the safety considerations of neurosurgical treatments in psychiatric contexts, there are two broad types of psychosurgical (another term for neurosurgical treatments targeted toward psychiatric conditions) approaches to consider: neuromodulation-based procedures (also known as functional or stereotactic neurosurgery) and ablative neurosurgery.

Safety considerations in neuromodulation-based neurosurgical procedures

Neuromodulation therapies, also known as stereotactic and functional neurosurgery, are minimally invasive surgical procedures that incorporate advanced neuroimaging techniques and technologies of the brain-computer interface to treat psychiatric disorders such as OCD, epilepsy, and eating disorders [65]. A few examples of functional neurosurgical therapies include deep brain stimulation, transcranial magnetic stimulation, and electroconvulsive therapy. Given the technologically reliant nature of neuromodulation-based surgeries, these therapies have been of more recent development and continue to be optimized with the advancement of new imaging and surgical tools.

Deep Brain Stimulation

Deep brain stimulation (DBS) has been used in the treatment of a multitude of psychiatric disorders such as treatment-resistant depression, obsessive-compulsive disorder, and anxiety disorders [14,17,24]. Much like many of other neuromodulation-based surgeries, DBS is considered minimally invasive and only requires a 10 cm incision in the skull, unlike other "open-skull" neurosurgical interventions [24]. Despite this seemingly low risk of complications, a study by Fenoy et al. demonstrated that DBS procedures still carry the potential for both short- and long-term consequences [52]. Specifically, the 2014 study found that out of 728 patients who underwent DBS, 0.5% faced asymptomatic intracerebral hemorrhage, 3.4% faced asymptomatic intraventricular hemorrhage, and 1.7% had decreased consciousness [52]. Regarding its long-term consequences, 1.1% of patients faced hardware discomfort and 1.4% had a decrease in the desired effect of the procedure [66]. Hence, although the likelihood of adverse outcomes is low, there are various considerations that practicing neurosurgeons utilizing DBS procedures need to take. Firstly, an important consideration for the surgical team would be to plan for potential complications that can arise and find ways to map the patient's neuroanatomy before surgical entry. This has become feasible with the advancements in Doppler and fMRI-based scanning tools [67]. More importantly, it is integral to find ways to monitor patient outcomes after they have left the hospital back into their homes. Although this aspect of patient monitoring is lagging in various aspects of medicine, new passive technologies such as smartphone pupilometers are being introduced to bridge this gap in neurosurgery [68]. Virtual "telehealth" appointments also offer more accessible means of contacting patients post-surgery and allow for the evaluation of patient concerns and outcomes [68].

Electroconvulsive Therapy

Electroconvulsive therapy (ECT) is a procedure that is commonly used in the treatment of severe obsessive-compulsive disorder, medication-resistant depression, aggression, and schizophrenia [69]. Since ECT was introduced as one of the first functional neurosurgical procedures, there have been a lot of optimizations that have drastically improved patient outcomes. In a recent study by Lippmann et al., 804 different routine ECT procedures were studied, and there were no severe negative outcomes documented [56]. Although there were minor side effects such as agitation and urinary stasis, when the surgical team employed certain "pre-procedural maneuvers" such as manually holding the chin and closing the patient's mouth when delivering electrical stimuli, these side effects decreased significantly. Additionally, given the reduced risk of adverse outcomes, ECT has shown to be a viable procedure in pregnant individuals who are negligent to take medications due to potential side effects [70]. The safe outcomes of ECT were further proven by a systematic review by Lima et al. [54], who demonstrated that using similar ECT maneuvers in adolescent patients resulted in highly effective treatment of psychiatric disorders as well as a significant reduction in adverse effects [71]. The most commonly associated adverse effects of ECT were short-term memory loss, headaches, muscle aches, confusion, nausea, and cardiovascular fluctuations, especially without optimizations such as anesthesia [70]. It may also pose rare risks such as prolonged seizures. Despite the common surgical risks associated with elderly patients, performing ECT on them (with comorbidities) has also shown to be relatively safe. Agelink et al. studied the risks associated with performing ECT on an elderly patient population with cardiovascular risk factors [57]. Their study indicated that further cardiovascular complications associated with ECT use were only seen in only one out of 24 patients [71]. Additionally, when compared with other procedures, there is no need for anesthesia [71]. Hence, ECT is a neurosurgical procedure that has shown to not only be effective at treating psychiatric disorders at a wide age range but has also evolved into a safe procedure with minimal to null risk of complications when performed correctly.

Transcranial Magnetic Stimulation

Transcranial magnetic stimulation (TMS) uses electromagnetic forces to stimulate specific regions of the brain through a changing magnetic field [72]. TMS is the least invasive functional neurosurgical approach. It has shown to be effective at treating epilepsy, depression, OCD, migraines, and smoking cessation [72]. Although TMS is a relatively recent innovation in the field of functional neurosurgery, there has been a growing amount of literature surrounding the safety and efficacy of the novel procedure. In a study exploring the use of interictal epileptic abnormalities, the authors found that TMS had negligent side effects and was safe for use in a wide age range like ECT. The same study reported that even with repeated and consistent TMS use in patients, the most common complications were relatively benign and rare, such as transient headaches, mild pain in the site of stimulation, and involuntary muscle contraction, which all resolved quickly [72]. This was further validated in another study that used TMS for the treatment of tic disorders and reported insignificant rates of complications [73].

Safety considerations in ablative neurosurgical procedures

While there are different ablative techniques used in psychosurgery with varying methods of action, they are all primarily used to selectively excise lesions of dysfunctional brain tissue in the hopes of recovering the connectivity within healthy brain matter. Various psychiatric illnesses have been approached through the lens of ablative neurosurgery, such as major depressive disorder (MDD), obsessive-compulsive disorder, and addiction. Although ablative neurosurgical procedures were deemed "crude" and lost credibility during the mid-1900s with the development of novel drug and neuromodulation therapies, recent advances in neuroimaging technology have brought back interest in these procedures [74].

Anterior Capsulotomy

Anterior capsulotomy (AC) is primarily a well-established procedure in the treatment of severe OCD. This procedure involves excising a lesion in the anterior limb of the brain's internal capsule, which is involved in the transmission of information to and from the cerebral cortex [75]. In a recent observational study-based literature review performed by Brown et al. [62], the use of AC as an OCD treatment plan projected a full response rate (measured by a clinically significant reduction in OCD symptoms after procedure) of 54%, with a transient and permanent rate of adverse event of 56.2% and 21.4%, respectively [76,77]. These adverse events were considered mild in nature and were characterized by headaches and pin-site swelling [77,78]. Another clinical outcome study also indicated AC to be more safe and effective when performed bilaterally on both sides of the brain [74,75]. This may be due to the important function of the anterior limb of the internal capsule (ALIC) in the limbic system, which requires the involvement of both hemispheres of the brain [78,79]. Additionally, in other larger research studies performed by Miguel et al. [66] and Gupta et al. [67], AC was indicated to be a viable, safe, and long-lasting operative procedure for patients with OCD as well as MDD. Most recently, MRI guidance was used to safely perform AC in four patients with refractory OCD and in one patient with MDD, successfully controlling symptoms for both indications [80]. Overall, not only has AC shown to be a viable procedure to effectively treat major psychiatric disorders such as OCD and MDD, but it also involves little to no risk to the patient.

Subcaudate Tractotomy

Subcaudate tractotomy is an ablative neurosurgical approach that involves selectively removing white matter tracts that connect the limbic structures and orbitofrontal cortex [81,82]. This procedure has changed significantly since its ideation due to the development and introduction of radiofrequency ablation procedures in medicine and neurosurgery. While the procedure was initially very invasive for patients and resulted in severe side effects such as motor dysfunction or paralysis, after adopting the RF approach, SCT has become relatively free from complications [83]. For example, in a study by Patel et al., out of over 660 individual cases of SCT, one patient passed away due to unrelated factors and 1.6% of patients experienced mild episodes of epilepsy as the most common complication [68]. Similar to other types of ablative surgical procedures (such as anterior capsulotomy), SCT is also a procedure that has been shown to have a higher level of safety when performed using image guidance [7]. Due to the microanatomical structures involved in the ablation process and the use of RF, it is integral for neurosurgeons to carefully plan the target location, and image guidance allows for this level of safety [84].

Limbic Leucotomy

Limbic leucotomy is a neurosurgical technique that involves removing sections of the anterior cingulate cortex and the subcaudate area of the patient's brain [79]. It was originally the primary surgical treatment modality for MDD and OCD, and it continues to remain a treatment option when patients do not respond to anterior cingulotomy alone [85]. During the initial period when LL was used in the late 1900s, not only were the results often unsatisfactory, but the procedure was also associated with major side effects such as hemiplegia or dystonia [80]. However, similar to other neurosurgical procedures, LL has had tremendous improvements in safety through the developments in neuroimaging, which have reduced the rate of complications substantially. Adverse events associated with LL are transient hallucinations, amnesia, and mania, which are all, as expected, associated with signaling through the limbic pathway. Although there are potential side effects associated with LL, most of these complications are quickly resolved and short-term [86]. The only complication that seems to be longer-term is abulia or the lack of motivation (although this is self-limited) [7]. Various other studies have also demonstrated the safe clinical use of LL. A study by Price et al. demonstrated that patients with schizophrenia who underwent LL not only had a significant reduction in symptoms but had minimal side effects that transiently diminished postoperatively [70]. Hence, LL is a procedure that has faced tremendous growth in safety and monitoring measures, which has allowed for effective treatments and positive patient outcomes.

There has been tremendous growth in the safety considerations and efficacy of psychiatric neurosurgical procedures with vast improvements in ablative techniques and advancements in modulation techniques. However, much like other procedures in medicine, the success in utilization of these procedures is not only reliant on the experience of the clinician but also on ensuring that the respective patient is an excellent candidate to endure the recovery process associated with the operation. Another important procedural consideration to ensure positive outcomes is following established standard-of-care approaches and avoiding risky techniques that may jeopardize the operation.

Future directions

Neurosurgery for psychiatric disorders is gradually moving toward a safer and more educated direction, through research and technological advancements around the world. An area of limited study, however, is the lack of insufficient reliable neural biomarkers that have the potential to catch psychiatric disorders at an earlier time point and allow for more minimally invasive surgical options. Potential electrochemical and physiological markers have been indicated by recent studies, and further research is warranted to validate the efficacy of using these markers in the clinical arena [81]. Scalp electroencephalography, stereo-EEG, magnetoencephalography, and electrocorticography are examples of local field potential data, which can be used in the comprehension of differences correlated to internal emotional states [82]. To analyze this data at a more rapid pace, artificial intelligence technologies are being developed and applied extensively [83].

Additionally, there is a lot of research on invasive neuromodulation that has engaged in open-loop neurostimulation. One of the examples is conventional DBS, which stimulates continuously. Low-frequency signals will trigger only nucleus accumbens stimulation during craving periods and were proven by recent obesity trials [83]. Moreover, studies have also shown that closed-loop neurostimulation combats treatment-resistant depression by focusing on brain regions using machine learning analyses. Private sector involvement has also participated in research for potential treatments for mental illness, with recent developments of brain-computer machines from companies such as Neuralink [3].

## Conclusions

The two most prevalent disorders targeted with psychosurgery are OCD and MDD. With advancements in neuroimaging techniques, knowledge of optimal targets in the brain for psychosurgery has greatly improved. Stimulation techniques such as DBS, VNS, TMS, and others have gained traction due to the possibility of implanting closed-loop systems. While neurosurgical management of psychiatric disorders is still considered experimental in many aspects, research and optimization of these procedures around the world have allowed them to gradually move toward a safer, more educated, and more effective direction.
